# Vibrational entropy of disordering in omphacite

**DOI:** 10.1007/s00269-023-01260-7

**Published:** 2023-11-27

**Authors:** Artur Benisek, Edgar Dachs, Michael A. Carpenter, Bastian Joachim-Mrosko, Noreen M. Vielreicher, Manfred Wildner

**Affiliations:** 1 Chemistry and Physics of Materials, University of Salzburg, Jakob-Haringer-Str. 2a, 5020 Salzburg, Austria; 2 Department of Earth Sciences, University of Cambridge, Downing Street, Cambridge, CB2 3EQ UK; 3 Institute of Mineralogy and Petrography, University of Innsbruck, Innrain 52, 6020 Innsbruck, Austria; 4 Institute of Mineralogy and Crystallography, University of Vienna, Josef-Holaubek-Platz 2, 1090 Vienna, Austria

**Keywords:** Order, Disorder, Pyroxene, Omphacite, Enthalpy, Entropy, Calorimetry, IR spectroscopy, Autocorrelation, Line broadening, Density functional theory

## Abstract

The cations of an ordered omphacite from the Tauern window were gradually disordered in piston cylinder experiments at temperatures between 850 and 1150 °C. The samples were examined by X-ray powder diffraction and then investigated using low-temperature calorimetry and IR spectroscopy. The low-temperature heat capacity data were used to obtain the vibrational entropies, and the line broadening of the IR spectra served as a tool to investigate the disordering enthalpy. These data were then used to calculate the configurational entropy as a function of temperature. The vibrational entropy does not change during the cation ordering phase transition from space group *C2/c* to *P2/n* at 865 °C but increases with a further temperature increase due to the reduction of short-range order.

**Supplementary Information:**

The online version contains supplementary material available at 10.1007/s00269-023-01260-7.

## Introduction

Omphacite is a pyroxene that can be described by the diopside (Di, CaMgSi_2_O_6_), hedenbergite (Hed, CaFeSi_2_O_6_), jadeite (Jd, NaAlSi_2_O_6_) and the acmite (Ac, NaFe^3+^Si_2_O_6_) components. Omphacites may have a long-range-ordered structure related to Mg/Al and Ca/Na cation ordering. According to Carpenter and Smith (1981), the long-range ordering is restricted to a compositional range between (Di + Hed)_40_ (Jd + Ac)_60_ and (Di + Hed)_60_ (Jd + Ac)_40_, not exceeding Ac_30_ content. This long-range-ordered structure was first described by Clark and Papike (1968). The ordering reaction causes a symmetry change from space group *C2/c* to *P2/n* (e.g. Matsumoto et al. 1975; Curtis et al. 1975; Rossi et al. 1983; Boffa Ballaran et al. 1998a), where the M1 site splits into M1 and M1(1), and the M2 site into M2 and M2(1), yielding h + k = odd reflections. Naturally ordered omphacites are often fully ordered on M1 (Mg/Al) but this is not the case for the M2 (Ca/Na) site. Here, a partially disordered state is observed (Clark and Papike 1968; Matsumoto et al. 1975; Curtis et al. 1975; Rossi et al. 1983). It was suggested that the best local charge balances are responsible for this partially ordering scheme (Rossi et al. 1983), which, however, could not be verified by simulation studies (Burton and Davidson 1988; Vinograd 2002).

The enthalpy of disordering (Δ*H*^dis^) was measured by solution calorimetry (Wood et al. 1980) and yielded ca. 8 kJ/mol when a naturally ordered omphacite was disordered at 1350 °C. Carpenter (1981) investigated the kinetics of the disordering reaction using experiments at various temperatures and for different durations (TTT analysis). Such a kinetic approach was also used to determine a Landau potential of *G* = 11.4 (*T*-1138) *Q*^2^ + 4317 *Q*^6^ (Carpenter et al. 1990a, b), where *Q* is the order parameter and *T* is the temperature in K. According to this potential, the maximum entropy of disordering (Δ*S*^dis^) is 11.4 J/mol/K, the maximum Δ*H*^dis^ is 8.7 kJ/mol and the transition temperature (*T*_c_) is 865 °C. This transition temperature was also predicted in static lattice energy calculations (Vinograd et al. 2007).

The difference of the vibrational entropy between ordered and disordered phases was investigated for a few metallic compounds like Ni_3_Al (e.g. Anthony et al. 1993), Cu_3_Au (e.g. Nagel et al. 1995) and Pd_3_V (e.g. Van de Walle and Ceder 2000). However, detailed descriptions of the temperature behaviour of this property are rare.

In this study, Δ*H*^dis^ and Δ*S*^dis^, including their configurational and vibrational parts, were investigated on samples equilibrated at different temperatures similar to our former studies on samples in the Cu-Au system (Benisek and Dachs 2015, 2018; Benisek et al. 2018). This allowed the determination of the enthalpy, the vibrational entropy and the configurational entropy of disordering as a function of temperature and allowed a comparison of the vibrational entropy behaviour between Cu_3_Au and omphacite. A correlation between line broadening in IR spectra and Δ*H*^dis^ was used to determine Δ*H*^dis^ of the different equilibrated samples (e.g. Carpenter and Boffa Ballaran 2001; Tarantino et al. 2003). It is based on the experience that mixing/disordering produces local strain heterogeneities that are indicative of a non-ideal mixing behaviour and can be characterised by the line broadening of IR spectra (Boffa Ballaran et al. 1998b).

## Experimental methods

### Starting material

The starting material was a naturally ordered omphacite with a grain size of ca. 0.1 mm from a kyanite eclogite (Harker no. 120853, Dept. of Earth Sciences, Cambridge University) from the central Tauern window (Austria). Its composition has been determined by Carpenter (1981) who calculated an averaged formula of (Ca_0.50_Na_0.48_)(Mg_0.45_Fe^2+^_0.06_Fe^3+^_0.01_Al_0.51_)Si_2_O_6_, i.e. Di_45_Hed_06_Ac_01_Jd_48_. The compositional range lies between Jd_43_ and Jd_52_ with up to Ac_04_ component (see Fig. 1 in Carpenter 1981). X-ray diffraction data indicate complete Mg/Al order on M1 and M1(1) and partial Na/Ca disorder in the range typical for other ordered omphacites (Carpenter 1981). Rossi et al. (1983) reported that the M2 site contains 0.75 Na + 0.25 Ca and the M2(1) site contains 0.25 Na + 0.75 Ca in omphacites from the Nybö eclogite.

### Disordering experiments

High-pressure experiments were performed in a Boyd-and-England-type piston cylinder apparatus (Boyd and England 1960). The pressure assembly consisted of a ½″ talc-Pyrex tube and a straight-walled graphite heater. The starting material was filled in Pt-capsules (inner diameter 3.0 mm, outer diameter 3.2 mm, length 10 mm) and dried at 393 K for 30 min in a drying oven. After removing the capsules from the oven, they were subsequently closed and welded shut to minimise the amount of surface water present in the capsule during the experiment. For each experiment, a capsule was placed in an inner container made of sintered MgO powder. The position of this container in the assembly ensures that the centre of the capsule is positioned at the hotspot of the graphite furnace during the experiment. Sample pressures were calibrated using the CsCl melting reaction (McDade et al. 2002), which is based on the CsCl melting curve established by Clark (1959) with an overall uncertainty in pressure determination of ± 0.1 GPa. Temperatures were determined with an uncertainty of ± 10 K using a Pt_90_Rh_10_—Pt (S-Type) thermocouple that was positioned at the bottom of the capsule. Runs were terminated by cutting off the power, which ensures that the temperature drops by several 100 K within seconds. An overview of the experimental conditions for each run is presented in Table 1.

**Table 1:** Conditions of heating experiments on natural omphacite (120853)

Experiment	Pressure (kbar)	*T* (°C)	*T* (K)	Duration (h)	XRD peak at 2*q* = 17.5°
120853	–	600	873.15	–	Strong
DB_4	18	850	1123.15	168	n.d
DB_2	18	920	1193.15	97.5	Absent
DB_3	18	950	1223.15	97.1	n.d
DB_1	18	1000	1273.15	91.7	n.d
DB_5	18	1050	1323.15	30.4	n.d
120853-dis	18	1100	1373.15	8	Absent
DB_6	18	1150	1423.15	28.9	Absent

It crystallised at 620 °C and 20 kbar (Holland 1979). Cation ordering was assumed to be frozen at 600 °C. Due to the small amount of material from the piston cylinder experiments, XRD investigations were not performed for all samples

### Calorimetry

Low-temperature heat capacities were measured with a relaxation calorimeter (Physical Properties Measurements System (PPMS) from Quantum Design^®^) between 2 and 300 K using a measuring technique described in Dachs and Benisek (2011). The sample powder (~ 10 mg) was placed into a cup made from an aluminium foil weighing ca. 5.5 mg. It was then closed at the top and pressed to a pellet (0.5 mm thickness, 3 mm in diameter).

### IR spectroscopy

The pellets for measuring the IR spectra were prepared following the procedure described in Boffa Ballaran et al. (1998b). Infrared spectra from the far-infrared region were collected with a Bruker IFS66v/S FT-vacuum spectrometer at the University of Vienna, using a Globar light source, a Mylar-6 µm beam splitter, and a DTGS-FIR detector. The spectra were measured with a spectral resolution of 2 cm^−1^ and averaged from 128 scans each. The autocorrelation method (Salje et al. 2000) was then used to investigate the line broadening due to the disordering.

### Calculations using the density functional theory (DFT)

Quantum–mechanical calculations were based on the DFT plane wave pseudopotential approach implemented in the CASTEP code (Clark et al. 2005) included in the Materials Studio software from Biovia^®^. The calculations used the local density approximation (LDA) for the exchange–correlation functional (Ceperley and Alder 1980) and norm-conserving pseudopotentials to describe the core–valence interactions. For the k-point sampling of the investigated unit cells, a Monkhorst–Pack grid (spacing of 0.05 Å^−1^) was used (Monkhorst and Pack 1976), and convergence was tested by performing calculations using a denser k-point grid. The structural relaxation was calculated by applying the BFGS algorithm (Pfrommer et al. 1997), where the convergence threshold for the force on an atom was 0.01 eV Å^−1^. The difference between calculated energy of mixing (Δ*E*^mix^) and enthalpy of mixing (Δ*H*^mix^) was assumed to be zero since the volume term *P* Δ*V* can be neglected (Benisek and Dachs 2020) for the mineral under investigation.

## Results

### Disordering experiments

The different heating experiments are listed in Table 1. The structures of some samples were investigated by powder X-ray diffraction focussing on the superlattice reflection h + k = odd ($\overline{1 }01$) at 2θ = 17.5° (Table 1). The results of the experiments show that at 920 °C, the omphacite becomes disordered within 97.5 h, in accordance with Carpenter (1981). The 850 °C/168 h experiment did not reach equilibrium conditions, but the sample was nonetheless investigated by low-temperature calorimetry to see the influence of cation disordering on the vibrational entropy below *T*_c_.

### Enthalpy

**Fig. 1:**
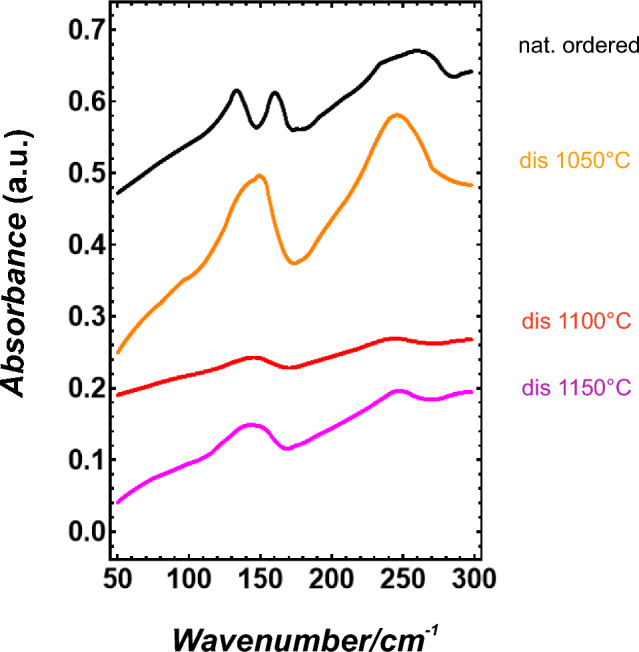
IR spectra of the investigated samples in the low wave number region

**Fig. 2:**
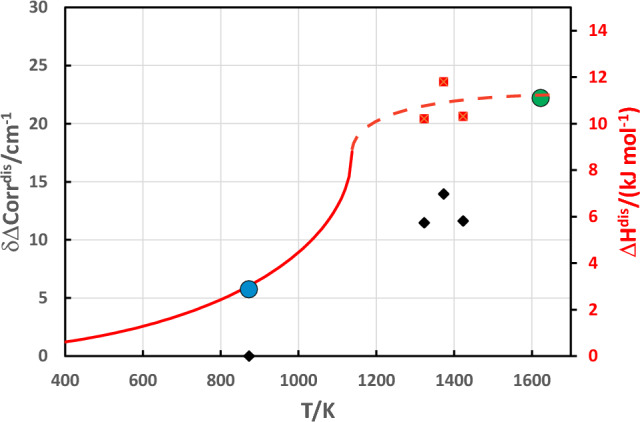
IR line broadening (δΔcorr^dis^) due to disordering (black diamonds) from the low wavenumber region and enthalpy (Δ*H*^dis^) of disordering (red squares) plotted against temperature. The solid line represents the tricritical Landau description of Carpenter et al. (1990a). The broken line is a visual guide for short-range behaviour. The blue circle is Δ*H*^dis^ of the naturally ordered sample calculated by the DFT method. The green circle at 1623 K is Δ*H*^dis^ measured by Wood et al. (1980) plus Δ*H*^dis^ of the naturally ordered omphacite sample

IR spectra of omphacite (Fig. 1) demonstrate a line broadening for the heated samples. The line broadening parameter (δΔCorr^dis^) was defined as

1 $$\delta \Delta {\text{Corr}}^{{{\text{dis}}}} = \Delta {\text{Corr}} -\Delta {\text{Corr}}^{{{\text{ord}}}} ,$$

where ΔCorr^ord^ is the autocorrelation parameter of the spectra from the naturally ordered sample. δΔCorr^dis^ from the low wavenumber region (50–300 cm^−1^) is plotted in Fig. 2. A correlation between the obtained δΔCorr^dis^ values and Δ*H*^dis^ is calculated for disordering the naturally ordered omphacite from the Tauern window at 1350 °C, using the Δ*H*^dis^ value of 8.28 kJ/mol from Wood et al. (1980), and yields Δ*H*^dis^/δΔCorr^dis^ = 0.671 kJ cm. Since the naturally ordered omphacite used in this study has a partially disordered state at the M2 and M2(1) site, it is characterised already by a Δ*H*^dis^ value, that can be calculated using the density functional theory. The energy of a cell containing 0.75 Na + 0.25 Ca on the M2 site and 0.25 Na + 0.75 Ca on the M2(1) site can be, therefore, compared to the fully ordered cell, which yields a Δ*H*^dis^ value of 2.81 kJ/mol for the naturally ordered omphacite (blue symbol in Fig. 2). Based on this information and the Δ*H*^dis^/δΔCorr^dis^ correlation, Δ*H*^dis^ values were determined and are plotted in Fig. 2.

The tricritical Landau potential *G* = 11.4 (*T*-1138) *Q*^2^ + 4317 *Q*^6^ from Carpenter et al. (1990a) was used to describe the long-range disorder. Above *T*_c_, short-range disorder increases Δ*H*^dis^ to a maximum value of Δ_max_*H*^dis^ = 11.1 kJ/mol at 1350 °C.

### Heat capacity

**Fig. 3:**
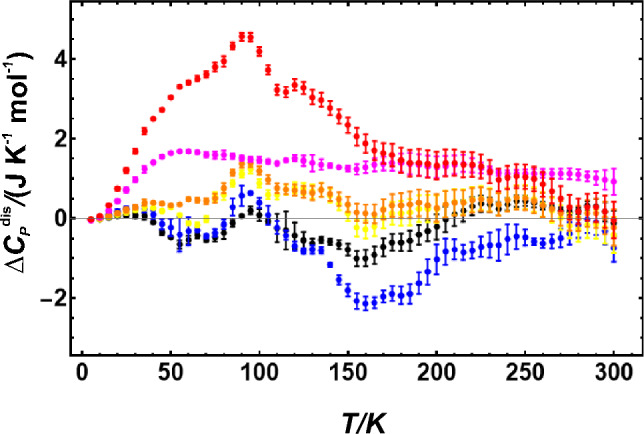
Heat capacity of disordering as a function of temperature. The samples were disordered at 920 °C (black), 950 °C (blue), 1000 °C (yellow), 1050 °C (orange), 1100 °C (magenta) and 1150 °C (red). Error bars represent 1 sd

The heat capacity of disordering (Δ*C*_*P*_^dis^), defined as the heat capacity of the naturally ordered sample subtracted from those of the disordered samples, shows a prominent deviation from ideal behaviour at around 100 K for samples equilibrated at high temperatures (Fig. 3). Such behaviour can be explained by weakened bonds due to the disordering reaction. It lowers the phonon frequencies, which are then excited already at lower temperatures.

### Entropy

**Fig. 4:**
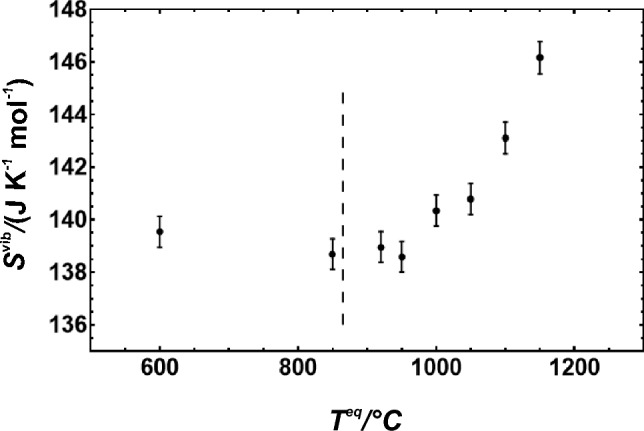
Measured vibrational entropy (*S*^vib^) at *T* = 298.15 K plotted against the temperature at which the cations were disordered. Broken line represents the transition temperature. Error bars represent 2 sd and were taken from Dachs and Benisek (2011)

If no magnetic disorder needs to be considered, as is the case in this study, the entropy of disordering of omphacite has two components, i.e. the vibrational and the configurational contributions. The vibrational entropy at room temperature (*S*_vib_^298.15^) is plotted as a function of disordering temperature in Fig. 4. The mean values of *S*_vib_^298.15^(*T*) decrease slightly between 600 < *T* < 950 °C but increase significantly above 950 °C. Short-range disordering has, thus, more influence on *S*_vib_^298.15^ than long-range disordering.

**Fig. 5:**
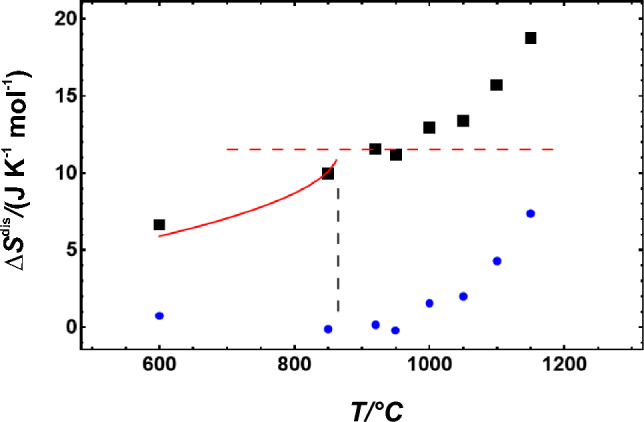
Entropy of disordering (Δ*S*^dis^) (black squares), as the sum of configurational entropy (red line), and vibrational entropy of disordering (blue circles) as a function of disordering temperature

Using the applied Landau potential, the heat capacity of the long-range disordering can be described. The configurational entropy can then be calculated from this heat capacity. The use of the tricritical Landau expression for cation ordering reactions is strictly speaking not correct. The entropy of such reactions depends not only on *Q*^2^ but also on higher order terms (Carpenter 1992). However, this expression should describe the configurational entropy change due to disordering reasonably well. It is plotted against temperature in Fig. 5. At *T*_c_, the experimentally determined configurational entropy almost reaches the theoretical value of two sites mixing. It can be calculated using the mole fractions *X*_A_ = *X*_B_ = 0.5 and the gas constant (R)

2 $${\text{S}}^{{{\text{config}}}} = \, - {\text{ 2 R }}\left( {X_{{\text{A}}} {\text{Ln}}\left( {X_{{\text{A}}} } \right) \, + X_{{\text{B}}} {\text{Ln}}\left( {X_{{\text{B}}} } \right)} \right),$$

which results in 11.53 J/(mol K) at the Di_50_Jd_50_ composition. The contributions from short-range disordering to the configurational entropy are not known. They, however, would shift them at *T* > *T*_c_ to higher values.

## Discussion

Thermodynamic disordering models normally describe the enthalpy and entropy changes for long-range disordering, i.e. the thermodynamic effect of disordering below *T*_c_. The vibrational entropy of disordering, measured in this study, is zero below *T*_c_ and increases at *T* > *T*_c_, which makes it difficult to implement it in existing thermodynamic models. Further investigations are necessary.

**Fig. 6:**
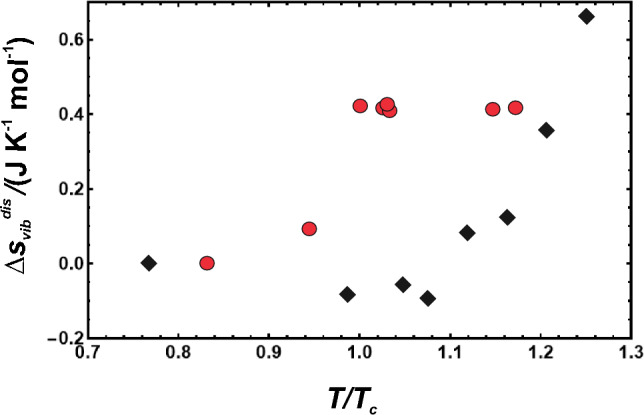
Monatomic vibrational entropy of disordering (Δ*S*_vib_^dis^) at 298.15 K plotted against normalised disordering temperature. The data from Cu_3_Au (red circles, from Benisek et al. 2018) are compared to omphacite (black diamonds, this study)

The temperature behaviour of the vibrational entropy of disordering (Δ*S*_vib_^dis^) can be compared with that of Cu_3_Au and Au_3_Cu, which are the only other phases that have been investigated in this regard. Since Δ*S*_vib_^dis^ of Cu_3_Au and Au_3_Cu behave similarly, the comparison will be with Cu_3_Au, because here much effort has been applied to decrease the experimental uncertainties (Benisek et al. 2018). For comparison with omphacite, we normalised the disordering temperatures with *T*_c_ and Δ*S*_vib_^dis^ with the atoms per formula unit (Fig. 6). The vibrational entropy of disordering in Cu_3_Au increases at *T*_c_. A further increase in disordering temperature, i.e. increase of short-range disordering, however, does not increase the vibrational entropy in Cu_3_Au in contrast to its behaviour in omphacite. The metallic compound Cu_3_Au has conduction electrons that contribute to the heat capacity and subsequently to the calorimetric entropy, which is not the case for omphacite. Could this difference be responsible for the differences seen in Fig. 6? The electronic entropy of Cu_3_Au was investigated by heat capacity measurements at very low temperatures by Martin (1976), who extracted the electronic contribution between 0.4 < *T* < 4 K, where this contribution is dominant. The data showed that the electronic heat capacity coefficients are *g* = 155.6 mcal/K^2^/mol for the ordered and 162.3 mcal/K^2^/mol for the disordered phase. The electronic heat capacity of disordering for Cu_3_Au is, thus, Δ_el_*C*_*P*_^dis^ = 6.7*T (in mcal/K/mol). Converting this value into SI units and integrating *C*_*P*_/*T* d*T* up to 680 K, which is the temperature of the Cu_3_Au phase transition, gives an electronic entropy of disordering of Δ_el_*S*^dis^ = 0.019 J/mol/K. This is only 5% compared to the vibrational entropy of disordering in Cu_3_Au, Δ*S*_vib_^dis^ = 0.4 J/mol/K, which was evaluated in the temperature range of 5 < *T* < 300 K and contains mainly lattice contributions (Benisek et al. 2018). We conclude, therefore, that the electronic contributions are not the reason for the difference observed in Fig. 6.

In a fully ordered omphacite structure, however, the chains are occupied by alternating Mg/Al and Ca/Na atoms, which produces optimal local charge balance within the pyroxene structure. Disordering in omphacites produces structures that are not locally charge balanced. This is very different to a simple solid solution like Cu_3_Au.

### Supplementary Information

Below is the link to the electronic supplementary material.

Supplementary file1 (PDF 105 kb)

Supplementary file2 (DOCX 15 kb)
