# Supplementary material for: Vibrational entropy of disordering in omphacite
Source: Phys Chem Miner. 2023 Nov 27;50(4):36. doi: 10.1007/s00269-023-01260-7 (PMC10682307; doi:10.1007/s00269-023-01260-7)

Figure S2 XRD-results from the study “Vibrational entropy of disordering in omphacite” published by A. Benisek, E. Dachs, M. A. Carpenter, B. Joachim-Mrosko, N. M. Vielreicher, M. Wildner, in Physics and Chemistry of Minerals.

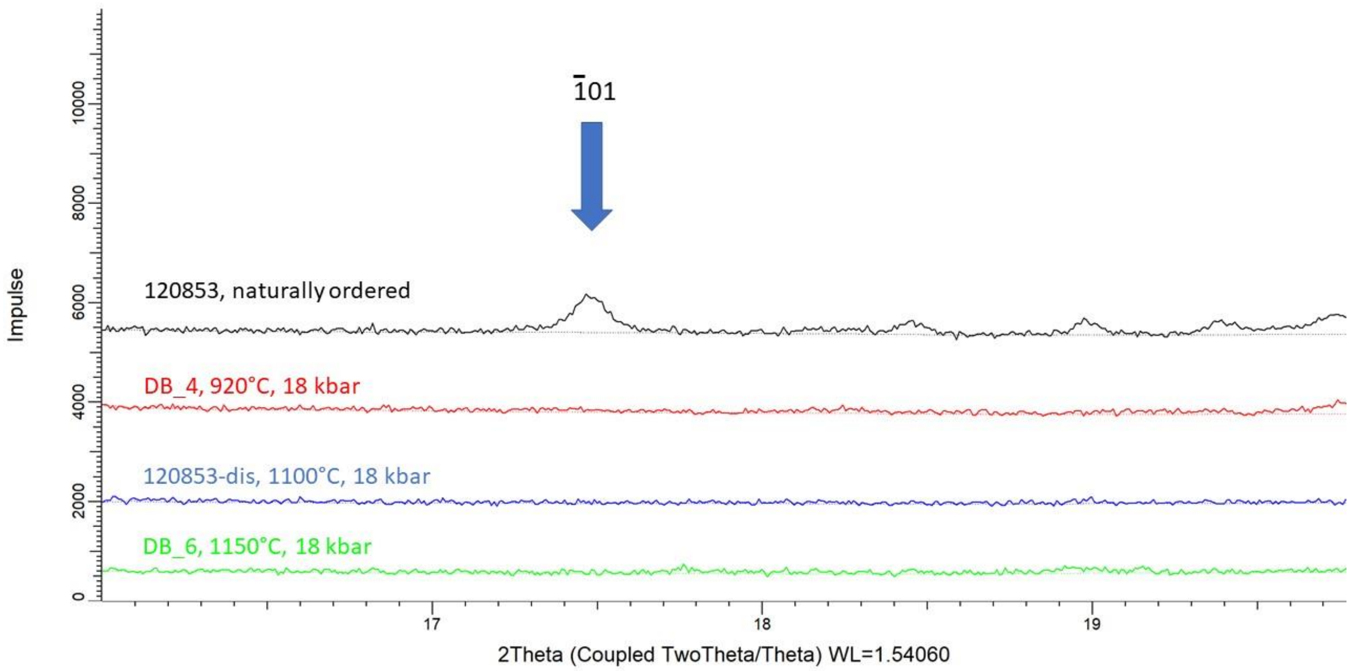

Supplement: Supplementary file 1 — Supplementary file1 (PDF 105 kb) [file 269_2023_1260_MOESM1_ESM.pdf]
