# Supplementary material for: Vibrational entropy of disordering in omphacite
Source: Phys Chem Miner. 2023 Nov 27;50(4):36. doi: 10.1007/s00269-023-01260-7 (PMC10682307; doi:10.1007/s00269-023-01260-7)
Supplement: Supplementary file 2 — Supplementary file2 (DOCX 15 kb) [file 269_2023_1260_MOESM2_ESM.docx]

**Table S1** Vibrational entropy at 298.15 °C, the autocorrelation parameters and the enthalpy of disordering as a function of disordering temperature from the study “Vibrational entropy of disordering in omphacite” published by A. Benisek, E. Dachs, M. A. Carpenter, B. Joachim-Mrosko, N. M. Vielreicher, M. Wildner, in Physics and Chemistry of Minerals.

| **Experiment** | **T (°C)** | **S_vib_^298.15^ (J mol^-1^ K^-1^)** |  | **Δcorr (cm^-1^)** | **δΔcorr^dis^ (cm^-1^)** | **Δ*H*^dis^**  **(kJ/mol)** |
| --- | --- | --- | --- | --- | --- | --- |
| 120853 | 600 | $139.54$ |  | 24.3 | 0 | 2.8 ^a)^ |
| DB_4 | 850 | $138.69$ |  |  |  |  |
| DB_2 | 920 | $138.96$ |  |  |  |  |
| DB_3 | 950 | $138.59$ |  |  |  |  |
| DB_1 | 1000 | $140.35$ |  |  |  |  |
| DB_5 | 1050 | $140.79$ |  | 35.8 | 11.5 | 10.2 ^b)^ |
| 120853-dis | 1100 | $143.10$ |  | 38.2 | 13.9 | 11.8 ^b)^ |
| DB_6 | 1150 | $146.15$ |  | 35.9 | 11.6 | 10.3 ^b)^ |

1. DFT value
2. Δ*H*^dis^ = 2.8 + 0.671*δΔcorr^dis^, see text
